# Whole Genome Complete Resequencing of *Bacillus subtilis* Natto by Combining Long Reads with High-Quality Short Reads

**DOI:** 10.1371/journal.pone.0109999

**Published:** 2014-10-16

**Authors:** Mayumi Kamada, Sumitaka Hase, Kengo Sato, Atsushi Toyoda, Asao Fujiyama, Yasubumi Sakakibara

**Affiliations:** 1 Department of Biosciences and Informatics, Keio University, Kohoku-ku, Yokohama, Japan; 2 Center for Information Biology, National Institute of Genetics, Mishima, Shizuoka, Japan; 3 Principles of Informatics Research Division, National Institute of Informatics, Chiyoda-ku, Tokyo, Japan; University of Strasbourg, France

## Abstract

*De novo* microbial genome sequencing reached a turning point with third-generation sequencing (TGS) platforms, and several microbial genomes have been improved by TGS long reads. *Bacillus subtilis* natto is closely related to the laboratory standard strain *B. subtilis* Marburg 168, and it has a function in the production of the traditional Japanese fermented food “natto.” The *B. subtilis* natto BEST195 genome was previously sequenced with short reads, but it included some incomplete regions. We resequenced the BEST195 genome using a PacBio RS sequencer, and we successfully obtained a complete genome sequence from one scaffold without any gaps, and we also applied Illumina MiSeq short reads to enhance quality. Compared with the previous BEST195 draft genome and Marburg 168 genome, we found that incomplete regions in the previous genome sequence were attributed to GC-bias and repetitive sequences, and we also identified some novel genes that are found only in the new genome.

## Introduction

New sequencing technologies, called second-generation sequencing, have changed the landscape of whole-genome sequencing by reducing the cost of sequencing and increasing throughput exponentially over first-generation Sanger [1] sequencing. Through this revolution of DNA sequencing, many scientists can now attempt whole-genome shotgun (WGS) sequencing of any organisms. Especially, microbial WGS sequencing holds great promise not only for enhancing clinical diagnostic study and public health microbiology [2], [3] but also for understanding production mechanisms of useful materials by microorganisms, such as the production of kanamycin and cephamycin by *Streptomyces clavuligerus*. Despite the great gains provided by second-generation instruments, complete high-quality genome sequencing still remains very difficult. A key reason for this difficulty is the *de novo* assembly of the genome from short reads [4]. Genome assembly is the process of reconstructing a genome sequence from sheared sequences generated as reads from DNA sequencing. Correctly recognizing repetitive sequences is needed for accurate *de novo* assembly of a genome, but most second-generation technologies produce relatively short reads, with maximum lengths of 300 bp using Illumina and 1000 bp using 454 platforms. These short reads make assembly increasingly difficult because repeats that are longer than the read length create gaps in the assembly, and an assembler cannot distinguish various repeats from short reads, which means that the regions flanking them can easily be misassembled [5].

In 2011, the PacBio RS, a real-time, single-molecule sequencer (Pacific Biosciences, Menlo Park, CA, USA) was released as the first commercial “third-generation” sequencing instrument. The PacBio RS sequencer produces very long reads with median lengths of a few thousand base pairs (average of approximately 8500 bp and longest reads exceeding 30,000 bp using the latest PacBio chemistry). Because long reads can easily handle complex regions such as repeats, PacBio long reads have the potential to provide accurate and improved genome assemblies [6], [7]. Actually, Chin et al. reported that the assembly of a microbial genome with PacBio reads generated the same number of contigs as chromosomes, and the assembled sequences showed more than 99.999% concordance with the Sanger-based reference genome [8].

In our previous study, the whole-genome sequence of *Bacillus subtilis* natto BEST195 was assembled and annotated [9]. *B. subtilis* natto is closely related to the laboratory strain *B. subtilis* Marburg 168 [10], which was the first sequenced genome of *B. subtilis* family and the best characterized gram-positive bacterium. *B. subtilis* natto produces “natto”, which is fermented soybean and is mainly consumed in Japan. Moreover *B. subtilis* natto synthesizes some effective products, such as poly-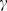-glutamic acid (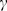-PGA), which is the major constituent of viscous material, in the process of natto production. Therefore, the genome sequence of *B. subtilis* natto has provided a great benefit to microbiology research and industrial studies. The *B. subtilis* natto BEST195 genome has been sequenced to a nearly completed state with very short reads of 36 bp in length using a comparative genome assembly method, which combines *de novo* assembly, reference assembly, and long polymerase chain reaction experiments to complete the assembled contigs. However, the BEST195 genome included some incomplete regions as gaps. Therefore, in this study, we sequenced the BEST195 genome using a PacBio RS sequencer and Illumina MiSeq with high-quality reads.

As described above, there are a lot of advantages of PacBio RS sequencing as typified by very long read length. However, the PacBio sequencer generates noisy reads that average only 82.1% to 84.6% nucleotide accuracy [11], [12], and the single-pass error rate of the PacBio RS is high (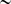
13%) compared with that of Illumina reads (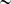
0.1%) [13]. Therefore, we should assemble genomes with PacBio reads carefully, and several different approaches and tools have been developed to overcome the above problem.

The earliest methods utilizing PacBio reads to assemble a genome improved genome sequences that were already assembled using short reads generated by other instruments such as Illumina and 454 [14], [15]. These methods align PacBio reads to an assembled genome and fill gaps within or between scaffolds based on aligned PacBio reads. Such an approach is useful when assembled scaffolds used only short reads of a certain length. In the wake of the above methods, several *de novo* assembly methods have been developed. They can be divided into two types in terms of error correction for PacBio reads, “hybrid methods” and “nonhybrid methods”.

First, hybrid methods such as PacToCA [16], LSC [17] and ECTools [18] correct read errors using high-quality short reads obtained by second-generation sequencing such as Illumina and 454. These methods map or align short reads or unitigs assembled from short reads to long reads first and then correct errors in long reads by making a consensus sequence. On the other hand, Cerulean [19] makes an assembly graph with short reads using an existing assembler based on the overlap or *de Bruijin* graph, and then it aligns long reads to the assembled contigs so that the assembly graph consists of only long contigs. To obtain the final assembled contigs corresponding to the scaffold, Cerulean adds short contigs to the assembly graph step by step.

Second, nonhybrid methods correct read errors only with PacBio long reads. The main methods of this type are hierarchical genome assembly process (HGAP) [8] and single-pass read accuracy improver (Sprai) [20]. PacToCA also can deal with PacBio reads to correct errors instead of short reads. These methods first correct errors in long reads by generating multiple alignments and making a consensus sequence, and then corrected reads are assembled using Celera Assembler [21].

Whereas all second-generation DNA sequencing have systematic sequencing errors, sequencing errors from the PacBio instrument occur randomly and independently. Therefore, most errors in PacBio reads can be corrected if the sequence depth is great enough and the consensus sequence from multiple alignments of reads has been done with accuracy. Actually, some studies have shown that nonhybrid assembly can generate longer and smaller numbers of contigs than hybrid methods [6], [8].

In this study, we took the following steps to assemble the *B. subtilis* natto BEST195 genome with PacBio reads and MiSeq reads. First, we employed nonhybrid assembly with only PacBio long reads to assemble the genome, and then we corrected the remaining errors via Illumina MiSeq at the finishing phase. After obtaining the complete genome sequence, we confirmed the incomplete regions in the previous genome. Moreover, we showed that the new complete BEST195 genome has the ability of refinement of the Marburg 168 genome sequence.

## Results

### Whole genome sequencing using PacBio RS platforms and Illumina MiSeq

Genomic DNA of *B. subtilis* natto was extracted from *B. subtilis* natto BEST195 [22] and WGS sequences were obtained using PacBio RS and Illumina MiSeq. Using 9 SMRT cells, 287,491 PacBio subreads totalling 594,815,476 nucleotides were recovered with a mean read length of 2,068 bp. On the other hand, 1,924,801 paired reads with a mean length of 231 bp were obtained after trimming and quality-filtering using Illumina MiSeq.

PacBio subreads and MiSeq paired-end reads were mapped to the previously published BEST195 genome sequence [9] using basic local alignment with successive refinement (BLASR) software [23] and Burrows-Wheeler alignment (BWA) tool [24], respectively. A total of 96.51% of the PacBio subreads and 93.30% of the MiSeq paired-end reads were mapped to the reference genome with 126- and 203-fold sequencing coverage across the entire genome. The results are summarized in Table 1.

**Table 1:** Summary of sequence reads.

Sequencer	Genomic DNA	Total # of reads	Average read length	Inferred coverage
PacBio RS	2.8 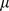 g	287,491	2,068	125.81
Illumina MiSeq	1.725 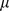 g	3,849,602	232	203.4

PacBio subreads and filtered and trimmed Illumina MiSeq reads were mapped to the previous BEST195 genome sequence using BLASR and BWA, respectively.

### *De novo* assembly from PacBio reads

Thirteen contigs were produced for *B. subtilis* natto BEST195 with the nonhybrid *de novo* assembly program Sprai [20] using only PacBio subreads. The total length of the assembled contigs was 4,207 kb, and that of five contigs with length greater than 10 kb was 4,140 kb. The length of the largest contig was 3,740,117 bases, which covers more than 90% of the previous genome (4,091,591 bases). To improve the assembly by removing sequence errors, we polished all contigs using Quiver [25] with PacBio subreads. After polishing contigs, the average number of mismatches between contigs and the previous BEST195 genome sequence was 14.25 per 100,000 aligned bases.

In our strategy, Illumina MiSeq reads were used at the finishing phase to polish the draft genome. On the other hand, short reads can also be used in the assembly phase with a hybrid assembler to improve the assembly results. To compare nonhybrid and hybrid assembly, we also assembled PacBio reads with Illumina MiSeq reads using a hybrid assembler, Cerulean [19]. Then, we obtained a total 40 contigs (38 contigs with a length greater than 10 kb), and N50 was 177,767 bases. Additionally, we assembled the genome using only Illumina MiSeq reads with SPAdes [26] and MaSuRCA [27]; then more than 100 contigs were obtained by both assemblers, and N50 was less than 110,000 bases. All assembly results are listed in Table 2. In our case, the nonhybrid assembler Sprai generated the best assembly results with a very small number of long contigs.

**Table 2:** Assembly results with PacBio subreads and Illumina MiSeq reads.

	Assembler	# contigs 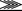 10 kbp	# contigs all	largest contig	N50	Total length 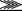 10 kbp	Total length all
Non-hybrid Assembly with PacBio	Sprai with Quiver HGAP	5	13	3,740,117	3,740,117	4,139,657	4,206,688
		5	7	3,192,554	3,192,554	4,131,147	4,145,559
Hybrid Assembly with MiSeq and PacBio	Cerulean	38	40	440,394	177,767	4,959,384	4,966,572
Assembly with MiSeq	SPAdes MaSuRCA	92 106	486 204	177,169 113,133	55,606 38,496	3,797,214 3,388,250	4,020,154 3,774,802

### Post-processing and finishing draft genome

BEST195 contains two plasmids pBEST195L and pBEST195S, and pBEST195S has been sequenced and reported as DNA data bank of Japan (DDBJ) accession number AP011542.1 in previous work [9]. Among 13 assembled contigs, we found one contig that completely matches pBEST195S with 100% identity, and we removed this contig. The redundancy check based on BLAST identified four redundant contigs from 12 contigs; that is, these contigs were contained within other contigs. After removing these four contigs, eight contigs were aligned to the previous BEST195 genome and *B. subtilis* Marburg 168 genome. Then, four contigs among the eight contigs were aligned to a large repetition region (roughly from 162 kb to 177 kb in the previous BEST195 and Marburg 168 sequence). We confirmed the contigs using the effective coverage information provided by Quiver output, and we excluded two contigs that were regarded as low-trust contigs. Finally, six contigs were merged into a single contiguous sequence using minimus2 in the AMOS software package [28], and one long scaffold and one singleton were obtained. The singleton was analyzed against the merged scaffold and was found in a scaffold as a subsequence. Because the *B. subtilis* genome is a circular DNA, we confirmed both ends and trimmed the overlapping region (334 bp), and then we obtained a genome sequence (4,105,391 bp). Additionally, the replication initiation site was adjusted based on the GC-skew plot and Marburg 168 genome sequence.

### Correction of the draft genome via PacBio long reads and Illumina short reads

In the merge process using minimus2, “N” is sometimes inserted if merged contigs have different bases. In our case, we found one “N” (at position 168,318) in the draft genome. We assigned the base as guanine by mapping PacBio subreads to the draft genome. PacBio subreads were mapped with a 97.8% mapping rate and mean coverage of 127.9, and 71.8% of mapped reads at that position were guanine.

Because PacBio RS sequencing errors occur randomly and independently [29], most errors can be removed with error-correcting algorithms for PacBio reads by making a consensus sequence. However, some errors might remain when it is difficult to make a consensus sequence, such as when two bases are equally present. We corrected the draft genome using high-quality and huge amount of Illumina MiSeq reads; 3.8 million Illumina MiSeq reads were mapped with a 96.33% mapping rate and 208.31-fold coverage using BWA. Then, 20 base changes, including three single nucleotide polymorphisms and 17 insertion/deletion (indel) changes were found using GATK [30]. The draft genome was corrected according to these 20 base changes.

Here, almost all of the detected base changes via MiSeq were the result of an indel. In the case of the PacBio RS sequencing, most sequence errors in PacBio reads are indels [13] because a missing or weak signal of nucleotide incorporation results in a deleted base, and a nucleotide that gives a fluorescence signal without being incorporated leads to insertions. Thus, the corrections via MiSeq were considered to be reasonable. Additionally, to avoid the false-positive corrections that arise from Illumina-specific sequence errors as reported in [31], we also confirmed all 20 positions by mapping PacBio subreads with BLASR. We found 32% 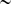 80% of mapped reads at each position have the same base as in MiSeq reads. Table 3 shows the details of each corrected position. Finally, we obtained the “complete” genome of 4,105,380 bp.

**Table 3:** Detail of corrected positions via MiSeq.

	base change			Mapping with MiSeq		Mapping with PacBio		
position	ref	alt	type	total reads	rate	total reads	rate (ref)	rate (alt)
166361	CA	C	del	88	99%	117	63%	32%
166597	T	TG	ins	25	100%	106	54%	46%
166783	TG	T	del	6	100%	101	57%	33%
171279	CG	C	del	42	95%	125	60%	33%
171308	A	AT	ins	63	84%	126	66%	34%
171362	G	A	snp	113	100%	128	7%	80%
171374	C	T	snp	124	100%	128	5%	78%
171760	G	A	snp	22	100%	102	8%	79%
526884	CA	C	del	400	97%	123	2%	79%
527199	AG	A	del	393	100%	107	57%	41%
527775	GT	G	del	227	92%	119	60%	34%
527806	TA	T	del	225	98%	118	58%	35%
3054380	CG	C	del	194	100%	168	58%	36%
3055055	CT	C	del	116	97%	156	53%	36%
3055082	TA	T	del	122	96%	156	49%	44%
3055240	TG	T	del	130	98%	156	60%	34%
3055338	CG	C	del	113	96%	156	56%	33%
3055553	GC	G	del	89	92%	144	58%	36%
3055595	C	CA	ins	113	96%	139	56%	44%
3056875	TG	T	del	44	100%	129	50%	42%

All PacBio RS reads and MiSeq reads were deposited in the read archive of DDBJ with accession number DRA001730, and the final genome sequence and annotation were updated with accession number AP011541.

### Gene annotation and genome comparison with *B. subtilis* Marburg 168

**Figure 1:**
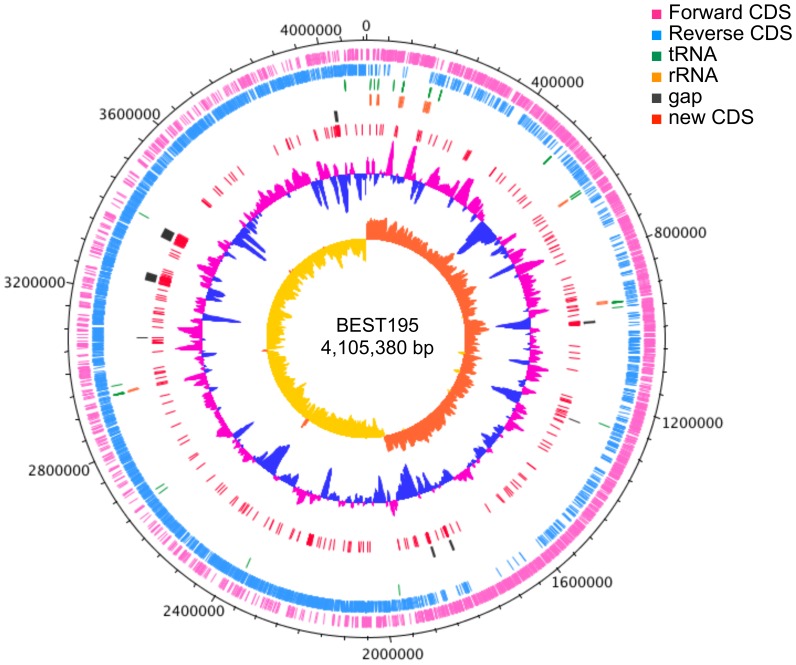
New BEST195 genome 4,105,380 bp. Pink and blue indicate forward and reverse genes, respectively. Black lines correspond to regions of gaps in the previous genome, and red lines indicate novel coding sequences (CDSs) found only in the new genome. The second inner circle from the centre displays the G+C content (window size  = 10,000 bp, step size  = 200), and the inner circle displays the GC skew. This genetic map was generated using DNAPlotter [32].

We annotate 4,589 genes in the new BEST195 genome, including 4,472 coding sequences (CDSs); 10 rRNAs for each 23 S rRNA, 16 S rRNA, and 5 S rRNA; and 87 tRNAs. Compared with the previous genome, 92.6% of 4,472 predicted CDSs (4,142) are annotated with the previous CDSs, and others (330) are present only in the new genome. Of the annotated CDSs, 95.8% (3,967) are annotated with a protein ID, which is issued by DDBJ/EMBL/GenBank for each amino acid sequence corresponding to a nucleotide sequence based on homology. Although the rest of the annotated CDSs (175) are located at almost the same positions as CDSs in the previous genome, some of them did not satisfy a criterion for homology because their length differed, and some have different predicted genes within the locus. The annotation of these genes was updated, and new protein IDs were issued. Figure 1 shows a genetic map of *B. subtilis* BEST195 chromosome generated using DNAPlotter [32].

Using the reciprocal best hit (RBH) method with BLASTx, 79.4% of 4,472 predicted genes (3,551) are orthologous to *B. subtilis* Marburg 168, and 15% of genes in Marburg 168 are deleted in BEST195. On the other hand, using the same procedures, we found seven genes that have orthologues in Marburg 168 and were present only in the previous genome. None of these orthologous genes were identified or predicted in the new genome to have the same length as in the previous genome. In the previous work to assemble the BEST195 genome, we used the Marburg 168 genome as a reference genome in the process of reference-guided assembly. Thus, it is possible that these genes have Marburg 168-like artificial sequences that are caused by the reference-guided process.

### Difference from the previous BEST195 genome

To confirm the differences between the new BEST195 genome and the previous BEST195 genome, we mapped three types of sequence reads to both genomes. One type of mapped reads is Illumina GAII, which was used to assemble the BEST195 genome in the previous study [9], and the other two are Illumina MiSeq and PacBio, which were used in this work. Table 4 shows the mapping results. PacBio reads (287,491) were mapped to the new genome and the previous genome with a mapping rate of 97.78% and 96.51%, respectively, and 3.8 million MiSeq reads and 6.9 million GAII reads were mapped to the new genome and the previous genome with a mapping rate of 96.34% and 93.30%, and 99.9% and 96.59%, respectively. For all types of reads, mapping rates were increased. Especially for MiSeq reads, the total number of reads with indels in the new genome was only one third as many as that in the previous genome.

**Table 4:** GAII, MiSeq, and PacBio mapping to the new genome and previous genome.

mapping with		new genome	previous genome
PacBio	Mapped reads (%)	97.78	96.51
	Coverage (fold)	127.9	125.8
	Mapping quality	241.28	239.32
Illumina MiSeq	Mapped reads (%)	96.34	93.30
	Coverage (fold)	209.4	203.4
	Mapping quality	58.47	57.87
	# base changes	6,878	24,242
Illumina GAII	Map reads (%)	99.09	96.59
	Coverage (fold)	59.6	58.3
	Mapping quality	57.72	57.40
	# base changes	1,552	4,307

Mapping quality is mean mapping quality of the mapped reads, and # base changes indicates the total number of reads with indels.

#### Gene

Most predicted genes were annotated with the previous genes, while the rest of genes were not annotated, that is, they are found only in the new BEST195 genome. Of the 330 novel CDSs, 58% did not have any BLAST hits against the non-redundant database, and 30% and 12% of them had homology with hypothetical proteins and other products such as transposase, terminase, and glycosyltransferase, which are expressed in the *Bacillus* genus, mainly in *B. subtilis*, *B. licheniformis* and *B. amyloliquefaciens*. Because some of them also have homology with genes in *B. subtilis* BEST195, it seems that they could not be identified in the previous genome because of highly similar sequences in other positions.

On the other hand, 233 previous CDSs were deleted from the new genome. Of these, 89% are hypothetical proteins, 6% of them are transposase, and 3% are ribosomal proteins. Additionally six tRNAs were also deleted from the new BEST195 genome. We confirmed these tRNAs using the above-described mapping results for three types of sequence reads. Then, we found that the deleted tRNAs (Met-Ser-Met) and (Trp-His-Gln) are present in other positions of the genome in the same order. Moreover, reads could not be mapped uniquely to regions where deleted tRNAs were present in the previous genome, while they could be mapped to the corresponding regions in the new genome. Therefore, they were misassembled in the previous genome.

**Figure 2:**
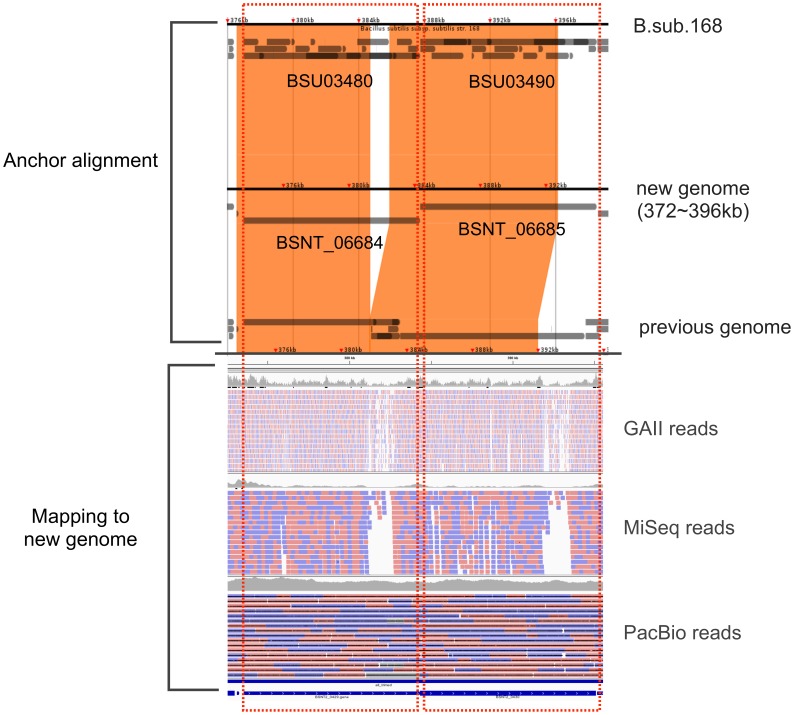
Example of novel orthologous genes. BSNT_06684 and BSNT_06685 are orthologous to BSU03480 and BSU03490, respectively. The upper part of this figure shows an anchor alignment within the new genome using Murasaki, a multiple genome comparison program [33], and the bottom part displays the mapping results of Illumina GAII reads, MiSeq reads, and PacBio reads.

Orthologous gene analysis with *B. subtilis* Marburg 168 allowed us to identify an additional five orthologous genes in the new genome. Figure 2 shows an example of these genes. Both orthologues have some regions where Illumina reads were not mapped uniquely, which means that these regions were difficult to assemble with Illumina reads, and these genes might have had imperfect regions in the previous genome. Thus, these genes are complemented in the new genome and orthologues in Marburg 168 could be confirmed.

#### Insertion sequence (IS)

Although *B. subtilis* Marburg 168 lacks typical ISs [10], many *B. subtilis* natto strains have various copies of IS, and previous work has demonstrated their presence and identified 40 transposase proteins. On the other hand, 56 transposase proteins were identified in the new genome. Transposases have a repeat-like sequence. Usually, repetitive regions are difficult to assemble from short reads. Therefore, identifying new transposases could be done using long reads.

#### Gap regions in the previous genome

The previous BEST195 genome contained 11 gap regions where bases could not be decided that ranged from about 20 bp to 20,000 bp. Three gaps were located in BSNT_00285 (16 s rRNA), BSNT_00287 (23 s rRNA), and BSNT_00625 (23 s rRNA), and they were filled with bases in the new genome (locus_tag: BSNT_06454, BSNT_06455, BSNT_06685). The other eight gaps were part of regions that were inserted into the Marburg 168 genome; that is, they are present only in *B. subtilis* natto. These regions were filled with bases, and we found 102 novel CDSs in the corresponding regions. Of the 102 CDSs, 57% and 26% have homology to hypothetical proteins and functional annotated proteins, such as transposase, transglycosylase, and glycosyl transferase, expressed in the *Bacillus* genus, respectively.

**Figure 3:**
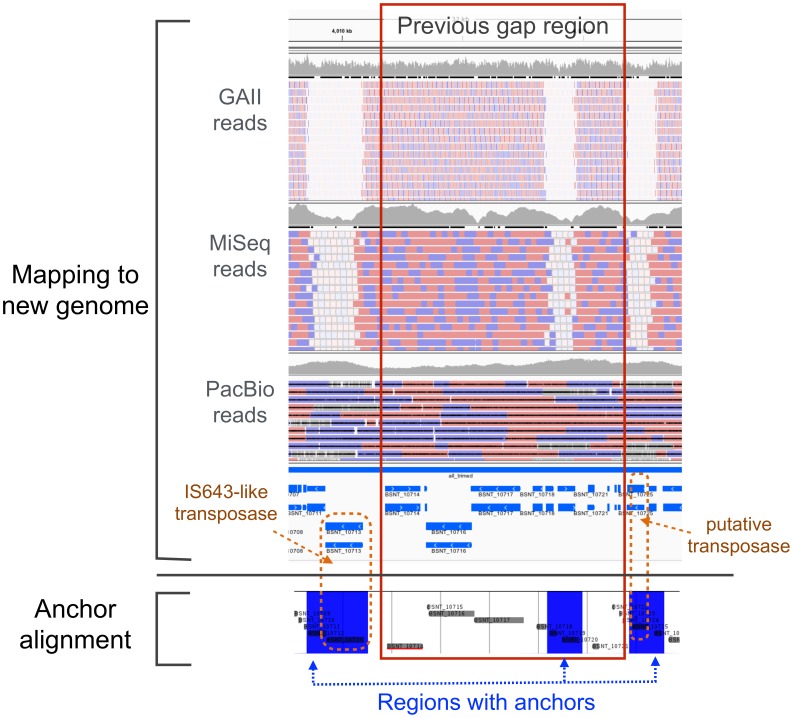
Example of one previous gap region. The region correspond to 4,003,725 bp to 4,007,944 bp in the previous genome. The upper part of this figure shows the mapping results of Illumina GAII reads, MiSeq reads, and PacBio reads. The bottom part displays an anchor alignment within the new genome using Murasaki, a multiple genome comparison program [33]. For the anchor alignment, blue regions represent aligned anchors, which means that the same subsequences are present in other positions of the genome. For the mapping results, reads coloured with white were mapped with mapping quality zero; that is, each read is not uniquely mapped to one position. Compared with the mapping results of Illumina reads, blue regions in the anchor alignment corresponded to positions where white reads were mapped. Additionally, both ends of the gap region include transposases with repetitive sequences.

To look at these gap regions in detail, we used the above-described mapping results for three types of sequence reads and the whole genome anchor alignments with themselves. An anchor shows a short well-conserved repetitive subsequence in the genome if the genome sequence is aligned to itself. We performed anchor alignments using Murasaki, which is a fast anchor-finding algorithm and multiple genome comparison program [33]. Here, we provide one example of gap regions in Figure 3. Figure 3 shows that the previous gap region included repetitive sequences and located between transposases. These regions are usually difficult to assemble with short reads. As with the above example, corresponding regions to the other seven gaps also have sequences that include repetitive anchors and the positions where Illumina reads were not mapped uniquely.

**Figure 4:**
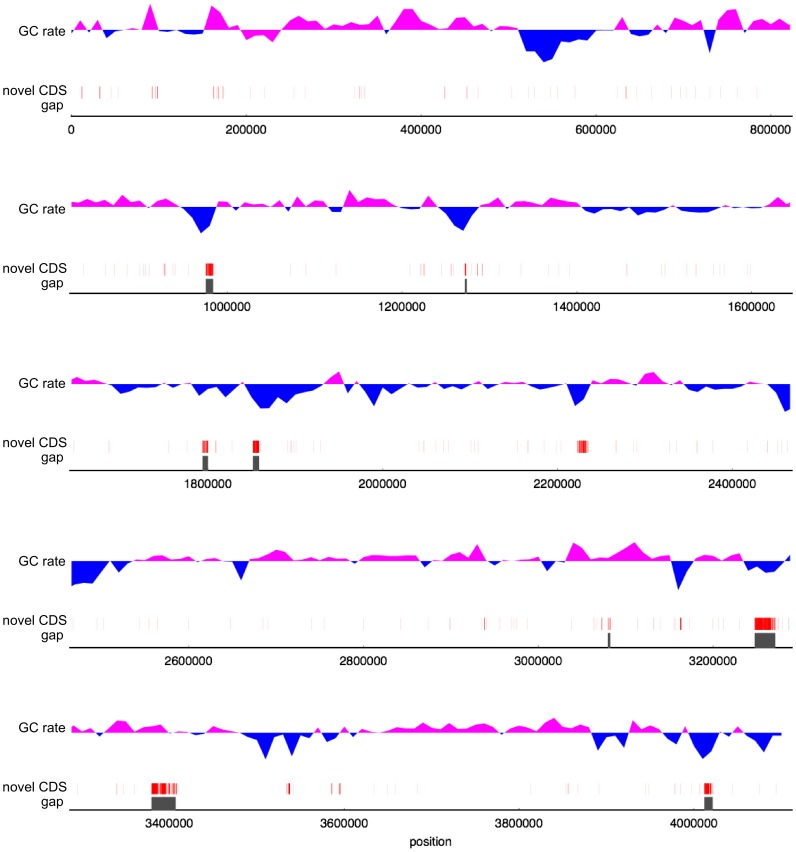
GC rate with novel CDSs and regions corresponding to gaps in the previous genome. GC rate was calculated with a 10,000-bp window. For the GC plot, pink and blue indicate an above-average GC rate and below-average GC rate, respectively. Red boxes indicate novel CDSs in the new genome, and grey boxes correspond to gaps in the previous genome.

Next, we confirmed the gap regions in terms of the GC rate. Figure 4 shows the GC rate for the new genome. The above-average GC rate and below-average GC rate are indicated in pink and blue, respectively. Additionally, regions corresponding to gaps are indicated in grey and CDSs found only in the new genome are red. As Figure 4 shows, six regions corresponding to gaps are located in low-GC regions, and another two regions are located in high-GC or neutral regions.

### Improving and refining the Marburg 168 genome

The *B. subtilis* has been a model for gram-positive bacteria and a typical member of the A+T-rich firmicutes. Joined by some 30 groups including US, Japanese, Korean and mainly European groups, the *B. subtilis* consortium sequenced and annotated the whole *B. subtilis* Marburg 168 genome, which is the laboratory standard strain of *B. subtilis*, in 1997 [10]. Since it had the drawback that the sequencing expertise was quite varied among the groups, the Marburg 168 genome was resequenced and reannotated using 454 sequencing technology in [34]. Moreover, we can expect the further refinement of the Marburg 168 genome sequence by the complete BEST195 genome utilizing the advantages of long reads. Compering the Marburg 168 genome sequence with the complete BEST195 genome sequence, there are 44,315 mismatches and 1411 indels between two genomes. The number of indels of length 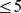 and length 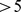 are 1255 and 156, respectively.

**Figure 5:**
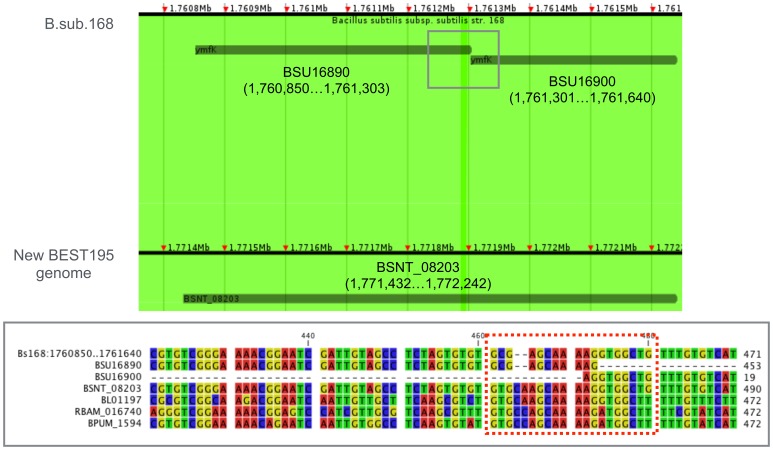
The improvable gene in the Marburg 168 genome by the complete BEST195 genome. The upper part of this figure displays an anchor alignment between the Marburg 168 genome sequence and the complete BEST195 genome sequence using Murasaki. The bottom part shows the alignment results of sequences of Marburg 168 genome, BEST195, and three relative species, *B. amyloliquefaciens*, *B. licheniformis*, and *B. pumilus*, using CLC Sequence Viewer. The substitution of C for T and deletions of C and A in the Marburg 168 genome sequence in red dash line box were thought to be the cause of separated genes, BSU16890 and BSU16900.

Figure 5 shows an example of an improvable gene. BSNT_08203 in BEST195 is functionally unknown gene of about 800 bases in length, and it was also found in three relative species as RBAM_016740 in *B. amyloliquefaciens*, BL01187 in *B. licheniformis*, and BPUM_1594 in *B. pumilus*. However, in the Marburg 168 genome, the gene corresponding to BSNT_08203 was identified in separate genes, BSU16890 (453 bases) and BSU16900 (339 bases). As shown in Figure 5, the Marburg 168 genome sequence has one substitution of C for T and two deletions of C and A at the end of BSU16890. Because this sequence region is well-conserved among the related species, these base changes in Marburg 168 were thought to be base errors and have caused the separation of the gene. Other base changes, which are regarded as base errors, were also found in the Marburg 168 genome sequence. Therefore, the Marburg 168 genome sequence is thought to be able to refine by comparing with the complete BEST195 genome.

## Discussion

In recent years, long reads produced by third-generation sequencing (TGS) platforms decreased the difficulty of genome assembly, making it possible to obtain almost single digit scaffolds in *de novo* assemblies of organisms with small genomes. Some genomes, such as those of bacteria or fungi, have been improved using TGS long reads. In this study, using PacBio RS long reads and Illumina MiSeq reads, we resequenced the *Bacillus subtilis* natto BEST195 genome, which was sequenced previously [9]. Although PacBio reads are often combined with high-quality short reads in the assembly process to reduce the high number of sequencing errors in the raw reads, some studies have shown that *de novo* assembly using only PacBio reads provides accurate results if the sequencing depth is sufficient. Thus, this study used only PacBio long reads to assemble the BEST195 genome with the nonhybrid assembler Sprai and MiSeq reads to polish the assembled draft genome. Then, we obtained a high-quality complete BEST195 genome (4,105,380 bp) without any gaps. Comparing the new BEST195 genome sequence with the previous sequence, we found 330 novel genes in the new genome including genes coding for transposases and genes that are orthologous to genes in the standard laboratory strain *B. subtilis* Marburg 168.

Almost all of the sequencing errors in PacBio reads were able to be removed by PacBio error correction processes because there were only 20 corrected positions via MiSeq. Most of these corrections were indels, which are the primary sequence errors of PacBio reads. Hence, the MiSeq polishing process is an effective and recommended way to obtain a high-quality draft genome sequence.

The previous BEST195 genome sequence had some incomplete regions as gaps, and these gaps were all filled with bases in the new genome sequence. Our investigation based on read-mapping and anchor alignment confirmed that almost all of the regions corresponding to gaps were GC-poor and included repeat-like sequences inside or around the region. Because these regions are usually difficult to assemble with short reads, the previous gap regions were thought to be caused by GC-bias and repetitive sequences. Moreover, the BEST195 genome contains some regions that are not found in the Marburg 168 genome, and all previous gaps were present in these regions. Therefore, the complete *B. subtilis* natto genome provides new insights concerning the mechanism of production in *B. subtilis* natto. On the other hand, the BEST 195 genome shares many sequence regions with the Marburg 168 genome, and the new BEST195 genome sequence has shown the ability of further refinements of the Marburg 168 genome sequence.

## Materials and Methods

### Reference and previous BEST195 genome

Because *Bacillus subtilis* BEST195 natto is closely related to *B. subtilis* Marburg 168, we used the Marburg 168 genome (GenBank NC_000964) for comparison analysis. In the previous study [9], the BEST195 genome was sequenced using the Illumina Genome Analyzer II. All sequence reads used in [9] have been deposited in the Read Archive at DDBJ with accession number DRA000001. The genome sequence was deposited as AP011541, but it already has been updated to the new genome sequence.

### Genomic DNA preparation and sequencing

The BEST195 strain is an isolate from Miyagino-based natto [22], and it has been used for various experiments [22], [35].

For PacBio RS sequencing, 2.8 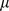
g of input genomic DNA was used for 10-kb library preparation. DNA was sheared with g-TUBE (Covaris Inc., Woburn, MA, USA) and purified using AMPure XP magnetic beads (Beckman Coulter Inc., Brea, CA, USA). Total 10
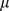
l library was prepared using PacBio DNA Template Prep Kit 2.0 (for 3–10 kb). SMRTbell templates were annealed using PacBio DNA/Polymerase Binding Kit 2.0. The PacBio DNA Sequencing Kit 2.0 and 9 SMRT cells were used for sequencing. Sequencing data collection was performed on the PacBio RS instrument for 90 min. Then, we filtered data with P_Filter  = 0.8 and MinReadLen  = 500 using pbh5tools (PacificBiosciences, Menlo Park, CA, USA) to obtain subreads.

Illumina libraries were prepared from 1,725 ng (115 ng/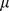
l×15 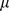
l) of BEST195 DNA using TruSeq DNA LT Sample Prep Kits (Illumina Inc., San Diego, CA, USA) as recommended. DNA was sheared with a Covaris instrument to 550 bases. The libraries were sequenced using 2×250 paired-end protocols on an Illumina MiSeq instrument (MiSeq Reagenet Kit version2 for 500 cycles). We obtained 32,429,140 reads with an inferred coverage of 1867x. All reads were trimmed to 10 to 240 bases, and the reads with a Phred quality below Q30 were filtered out using FASTX-Toolkit (http://hannonlab.cshl.edu/fastx_toolkit/).

### Genome assembly with PacBio RS reads

PacBio subreads were assembled with Sprai version 0.9.5 [20] with default settings. We used Cerulean [19] for a hybrid *de novo* assembly using PacBio reads and Illumina MiSeq reads. To prepare an input assembly graph for Cerulean, we employed ABySS [36], which is an assembler for short reads, and used k-mer  = 62. Additionally, we assembled only Illumina MiSeq reads using SPAdes [26] and MaSuRCA [27].

To significantly reduce the remaining base errors including indels in the draft assembly, Sprai recommends using Quiver [25] to polish the assembled contigs. Quiver is a base-calling program developed by PacificBiosciences. According to Sprai documentation, PacBio raw reads are aligned against the assembled contigs using “pbalign”, which is a tool for aligning PacBio reads to some genome developed by PacificBiosciences. Then, consensus sequences are generated by running Quiver.

### Scaffolding and polishing the draft genome using Illumina short reads

To obtain a long draft genome from the assembled contigs, we used scaffolds according to the following steps. First, BEST195 contains two plasmids pBEST195L and pBEST195S, and pBEST195S has been sequenced and reported as DDBJ accession number AP011542.1 in a previous study [9]. Thus by using BLAST alignment against 13 contigs, we found one contig that completely matched the pBEST195S sequence with 100% identity. Next, we checked the redundancy of the assembled contigs using BLAST and a Sprai module (check_redundancy.pl), and 4 contigs were identified as redundant. Therefore, we removed the contig that matched the plasmid and four seemingly redundant contigs manually.

Because there were still small and similar contigs, we aligned the remaining eight contigs to the previous BEST195 genome and *B. subtilis* Marburg 168 using BLAST, and we found that four of eight contigs were aligned to regions with repeated rRNA. To examine the quality of the four contigs, we utilized the effective coverage information provided by the Quiver output. In the Quiver process, PacBio raw reads are aligned to each contig, and for a region with few aligned reads, Quiver assigns a lower-case character to indicate “no effective coverage regions”. In our case, two of four contigs included many lower-case characters, and we removed these two contigs. Then we merged the remaining six contigs using minimus2, which is a pipeline designed for merging sequence sets and it provided in AMOS version 3.1.0 [28].

Because *B. subtilis* has circular DNA, we confirmed both ends of the genome using NUCmer and show-coords, which are modules in Mummer 3.23 [37], and we trimmed overlapping sequences (334 bases). In circular bacterial chromosomes, the origin and terminus of DNA replication are predicted using GC skew [38]. GC skew is a nucleotide composition bias of guanine and cytosine defined by 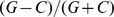, where 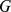 and 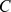 represent the frequency of guanine and cytosine. The first base of the *B. subtilis* Marburg 168 genome was adjusted to match the origin of replication. Thus, we identified the origin based on the calculated GC skew and Marburg 168 genome, and we adjusted the origin of the genome.

### Correction of the draft genome using PacBio reads and Illumina MiSeq reads

To fill the undetermined bases that arose in the merging process by minimus2, we mapped PacBio reads to the draft genome using BLASR [23] and manually decided the bases that mapped at the position with IGV [39]. Next, to refine the draft genome with Illumina reads, we mapped Illumina MiSeq reads that were filtered for high quality (Q = 30) to the draft genome using BWA [24], and identified base changes using GATK [30]. To identify base changes, we used VariantFilteration, which is a GATK tools for hard-filtering variant calls, with the parameters set to DP 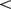 3 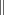 QUAL 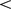 20.0 and QD 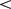 5.0 for SNV and MQ0 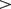 4 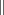 DP 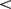 3 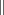 QUAL 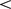 20 for indel. After the correction by MiSeq reads, we validated these modifications by a manual check with IGV based on the mapping results with PacBio reads via BLASR.

### Gene prediction and gene annotation

To predict gene coding regions in the new genome, we used the bacterial genome annotation service xBASE [40]. xBASE employs Glimmer [41], tRNAScan-SE [42], and RNAmmer [43], and it produces a combined result. Although xBASE automatically annotates each gene with a pre-installed user-selectable reference genome, we applied BLASTx [44] against the original database for previous BEST195 genes and annotated each gene based on similarity scores.

We used the same locus_tag and protein_id as the previous gene if a gene was present in exactly the same locus and had high similarity with the previously annotated gene; otherwise we provided a new locus_tag. In cases when a gene was deemed to be the same as the previous gene in terms of position and similarity score, we annotated it as the old_locus_tag, which indicates the previous locus of the gene.

To identify orthologous genes of *B. subtilis* Marburg 168, we used the RBH method with BLASTx. In this method, a gene 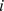 in species A is an RBH of gene 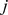 in species B if a query of species A with gene 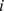 yields gene 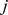 as the top hit, and a reciprocal query of species B with gene 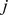 yields gene 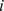 as the top hit.

### Differences between the new genome and the previous genome

To confirm differences between the new BEST 195 genome and the previous BEST195 genome, we mapped three types of reads; Illumina GA II, which was used in the previous study [9], Illumina MiSeq, and PacBio reads. Illumina GA II reads and MiSeq reads were filtered using FASTX-Toolkit with q = 10 and p = 90, and q = 30 and p = 100, respectively. 287,491 PacBio subreads were mapped using BLASR [23], and 3,849,654 Illumina MiSeq reads and 6,856,498 Illumina GAII reads were mapped using BWA [24]. For each mapping result, coverage, mapping quality, and the number of base changes were calculated using Qualimap 0.7.1 [45].

We detected similar repetitive subsequences within the new genome based on anchors, which are well-conserved sequences usually between two genomes. Anchors were calculated using Murasaki, which is a fast anchor-finding algorithm [33]. The GC rate of the genome was calculated with a 10,000-bases window, and is illustrated in Figure 4 by two colours based on the average GC rate for overall the new genome (43.5%).

### Improving and refining the Marburg 168 genome

To assess the differences between the *B. subtilis* Marburg 168 genome sequence and the new BEST195 genome sequence, we used QUality ASsesment Tool (QUAST) version 2.2 [46]. Moreover, we compared two sequences using an anchor alignment with Murasaki [33], and we performed and visualized multiple sequence alignment for relative species using CLC Sequence Viewer 7.0.2 (CLC Inc., Aarhus, Denmark).

### Data deposition

All reads have been deposited in the Read Archive at DDBJ with accession number DRA001730, and the final genome sequence and annotations have been deposited in DDBJ as an updated version with accession numbers AP011541. The determined genome sequence of *B. subtilis* natto, gene predictions and annotations with the three *Bacillus* species comparisons are available on our Natto genome browser http://natto-genome.org/.
